# The Imprinted PARAFILM as a New Carrier Material for Dried Plasma Spots (DPSs) Utilizing Desorption Electrospray Ionization Mass Spectrometry (DESI-MS) in Phospholipidomics

**DOI:** 10.3389/fchem.2021.801043

**Published:** 2021-12-10

**Authors:** Jiansong Chen, Yue Hu, Congxiang Shao, Haiyun Zhou, Zhiyue Lv

**Affiliations:** ^1^ Instrumental Analysis and Research Center, Sun Yat-sen University, Guangzhou, China; ^2^ Key Laboratory of Tropical Disease Control (Sun Yat-sen University), Ministry of Education, Guangzhou, China; ^3^ Provincial Engineering Technology Research Center for Biological Vector Control, Guangzhou, China; ^4^ Key Laboratory of Tropical Translational Medicine of Ministry of Education, Hainan Medical University, Haikou, China; ^5^ Department of Gastroenterology of the First Affiliated Hospital, Sun Yat-sen University, Guangzhou, China

**Keywords:** carrier material, desorption electrospray ionization mass spectrometry, dried plasma spots, phospholipidomics, parafilm

## Abstract

The application of desorption electrospray ionization mass spectrometry (DESI-MS) and dried blood spot (DBS) sampling has been successfully implemented several times. However, the difficulty of combining DBS sampling with DESI-MS is still the carrier material used for the blood samples. In this study, a new, easily obtained, and cost-effective carrier substrate for dried plasma spot (DPS) sampling and DESI-MS analysis and its application in phospholipidomics studies was described. First, the effects of several carrier materials, including cellulose-based materials (31 ET paper and filter paper) and non-cellulose-based materials (PARAFILM and its shape-modified material, PTFE-printed glass slide and polyvinylidene fluoride film), were tested. Second, a method combining DPS sampling with DESI-MS for phospholipidomics analysis was established, and parameters affecting compound signal intensities, such as sample volume and sprayer solvent system, were optimized. In conclusion, the total signal intensity obtained from shape-modified PARAFILM was the strongest. The suitable plasma sample volume deposited on PARAFILM carriers was 5 μl, and acetonitrile (ACN) was recommended as the optimal spray solvent for phospholipid (PL) profiling. Repeatability (87.5% of compounds with CV < 30%) and stability for data acquisition (48 h) were confirmed. Finally, the developed method was applied in phospholipidomics analysis of schistosomiasis, and a distinguished classification between control mice and infected mice was observed by using multivariate pattern recognition analysis, confirming the practical application of this new carrier material for DPS sampling and DESI-MS analysis. Compared with a previously reported method, the rapid metabolomics screening approach based on the implementation of DPS sampling coupled with the DESI-MS instrument developed in this study has increased analyte sensitivity, which may promote its further application in clinical studies.

## Introduction

Phospholipids (PLs), as the major components of the cell membrane, exist in both eukaryotic and prokaryotic organisms (Hanahan, 1997); PLs play important roles in controlling and regulating cellular function and participate in many cellular biological processes (Subramaniam et al., 2011). Recent studies have shown that the concentrations of PLs in blood are associated with the progression of multifarious diseases, such as cancer (Viswanathan et al., 2017), Alzheimer’s disease (Bazinet and Laye, 2014), obesity, diabetes mellitus and cardiovascular disease (Meikle and Summers, 2017). Over the past few decades, PLs have generally been obtained through liquid-liquid extraction methods (Bligh and Dyer, 1959) or protein precipitation methods (Sarafian et al., 2014) to detect the metabolic fluctuation in PLs *in vivo*. These methods both include deproteinization, solvent extraction, centrifugation, and other preprocessing steps, followed by time-consuming chromatographic separation. However, these procedures limit sample throughput and inevitably lead to the loss of metabolites. Thus, it is necessary to develop a high-throughput phospholipidomics method to profile the changes in PLs in organisms.

Desorption electrospray ionization (DESI), an ambient technique applied in mass spectrometry (MS), allows for *in situ* analysis with little to no sample pretreatment (Takats et al., 2004). Owing to these compelling advantages, direct surface sample analysis in phospholipidomics (Eberlin et al., 2011; Gross, 2017) has received a great deal of attention. The electrospray ionization (ESI) mechanism of DESI has been reported primarily by Cooks and coworkers and was previously suggested to follow the “droplet pickup” model, in which droplet pickup of analytes by charged droplets impacts the sample surface (Venter et al., 2006). In detail, highly charged microdroplets in the atmosphere form a thin layer of solvent on the sample surface by bombardment, and analytes dissolve into a “solvent thin layer”, which can be extracted (Costa and Cooks, 2008). Subsequent collisions of droplets occur, second-generation microdroplets are formed, and analytes desorb *via* momentum and transfer from the sample surface into the gas phase during impact (Kebarle and Verkerk, 2009).

Blood is one of the most significant body fluids for the research of various diseases; however, because liquid samples should be spotted on a substrate and dried before DESI-MS analysis, new sampling approaches are being developed to satisfy the direct analysis of body fluids. To date, dried blood spot (DBS) sampling has been the most suitable technique. DBS sampling was first reported as an alternative blood sample collection approach in 1963 and was then successfully introduced to neonatal screening for inborn errors of metabolism (Guthrie and Susi, 1963). The obvious advantage of DBS sampling is the small collection volume of blood. Thus, the invasion of DBS sampling is minimal and painless. On the other hand, DBS sampling is more convenient for sample processing and storage than liquid biological matrices. As mentioned above, DBS sampling has become a very useful tool for metabolic disorder diagnosis at present, especially in biochemistry studies (Li and Tse, 2010), pharmacokinetics (Saini et al., 2018), toxicokinetics (Raju et al., 2016), therapeutic drug monitoring (Gaissmaier et al., 2016; Zheng et al., 2016), and newborn screening for inborn errors of metabolism (Wilcken and Wiley, 2008). However, since blood with a relatively high haematocrit (HCT) level will spread less than that with a low HCT level, the HCT level is one of the factors that must be considered in DBS sampling. The distinction of spot diffusion leads to a substantial difference in DBS area, which in turn results in discrepant sample proportions during DESI-MS scanning. To date, it is quite challenging to eliminate the variance caused by HCT since the HCT level is affected by many factors. Therefore, using plasma instead of whole blood for the generation of dried matrix spots could be more reasonable, which led to the development of dried plasma spot (DPS) sampling (Barfield and Wheller, 2011).

Another challenge of DESI-MS analysis with DBS is the choice of carrier substrate. Various attempts have been made to produce the highest signal and the least ionization suppression (Leuthold et al., 2006; Wiseman et al., 2010). An increasing number of commercial or homemade substrates, including silicon-based materials, such as glass slides (Pasilis et al., 2007; Manicke et al., 2008) and glass slides coated with heavy Teflon coating (HTC) material (Mattarozzi et al., 2012), and thin-layer chromatographic plates (Kauppila et al., 2006), porous silicon chips (Schwab et al., 2014), and carbon-based materials, such as polymethyl methacrylate (Volny et al., 2008), PTFE (Manicke et al., 2008; Volny et al., 2008), PTFE-printed glass (Manicke et al., 2008), paper (Manicke et al., 2008) and porous Teflon membranes (Rosting et al., 2013; Sero et al., 2015), have been investigated for DESI-MS analysis. From these studies, we have concluded that an electrically nonconductive material could enhance the response of DESI-MS since the material preserves charges by avoiding neutralization of the incoming ion plume at the surface. In addition, charges can be better focused with a nonconductive material than with a conductive material, along with higher gas pressure and less spray tip-to-surface distance (Bianchi et al., 2020). More importantly, a non-cellulose-based material with a porous or rough surface could also improve ionization efficiency due to a reduction in sample spread and absorption.

To the best of our knowledge, applying DESI-MS analysis in DPS sampling to analyse metabolites directly, especially in biomarker discovery and early clinical diagnosis, has rarely been investigated. Therefore, the aim of this study was to determine a suitable carrier material for DESI-MS to increase the application reach of DESI-MS. In this study, easily obtained and cost-effective carrier materials for DPS, including cellulose-based and noncellulose-based materials, were evaluated. Then, a method for PL profiling by DESI-MS with DPS sampling was established, and parameters affecting the signal intensities of PLs in DPS, such as sample volume and sprayer solvent, were optimized. Ultimately, the optimal method was applied to investigate the changes in PLs in mice infected by *Schistosoma japonicum* (*S. japonicum*) to assess the feasibility of the method.

## Materials and Methods

### Materials and Samples

Acetonitrile (ACN), methanol (MeOH) and isopropanol (IPA) of LC-MS grade were obtained from Fisher Corporation (Hampton, NH, United States). Leucine enkephalin (LE) and MS-grade formic acid (FA) were obtained from Sigma-Aldrich (St. Louis, MO, United States).

The cellulose-based materials included 31 ET papers and double ring quantitative filter papers (Whatman, Piscataway, NJ, United States). The non-cellulose-based materials included PARAFILM^®^ M film (PM-996) (Bemis, Neenah, WI, United States), polyvinylidene fluoride (PVDF, 0.45 μm) film (Merck Millipore, Bedford, MA, United States) and PTFE-coated glass slides (Prosolia, Zionsville, IN, United States).

Ten specific-pathogen-free 8-week-old female BALB/c mice were provided by the Animal Experiment Center at Sun Yat-sen University (Guangzhou, China) and were divided equally into two groups. After the mice acclimated to the new environment, five were infected with 30 ± 2 *S. japonicum* cercariae per individual, while the other mice were left uninfected and served as controls. At 42 days post-infection (dpi), all mice were sacrificed by chloral hydrate asphyxiation and cervical dislocation. For each mouse, whole blood was drawn from orbital veins, transferred to a vacuum blood collection tube containing EDTA-K2, transferred into Eppendorf tubes, centrifuged at 1,500 rpm for 5 min at 4°C to collect the plasma, and frozen subsequently at -80°C. All animal experiments were approved by the local ethical committee.

### Preparation of Substrate Holders and DPS Samples

Six types of substrate holders, including modified PARAFILM, original PARAFILM, PVDF film, PTFE-printed glass slides, 31 ET, and filter papers, were fixed on microscope glass slides (76 mm*26 mm) using double-sided tapes (Supplementary Figure S1). The modified PARAFILM was created by imprinting a certain number of circular grooves with 4 mm in diameter and 0.1 mm in depth on the surface. The spacing of each groove was 4 mm (Supplementary Figure S2).

Three different volumes of plasma samples (2, 5 and 10 μl) were deposited on the different carrier materials using a calibrated 1–10 μl range micropipette (Eppendorf, Hamburg, Germany). It was ensured that the same volume of sample covered the carrier materials uniformly. Afterwards, the blood spots were dried at room temperature in a desiccator for 24 h. Each experimental condition was performed in triplicate, and the average peak intensity was used to assess the effectiveness of the sample preparation method.

### Instrumentation

All experiments were performed on a SYNAPT G2-Si high-definition mass spectrometer (Waters, Milford, MA, United States) coupled with a Waters modified two-dimensional DESI stage source (Prosolia, Zionsville, IN, United States).

DESI-MS experiments were carried out with an applied voltage of 5 kV in positive ion mode, and the operating conditions were set at a flow rate of 10 μl/min using a Harvard syringe pump. Nitrogen as nebulizing gas was delivered at 8 bars. The source temperature was set at 150°C, and the cone voltage was 45 V. Rhodamine-6G (a red marker, with a mass/charge (*m/z*) of 443 in positive mode) was used to determine the initial geometrical parameters of DESI-MS: horizontal distance from the MS inlet to the TaperTipTM emitter, 4–5 mm; vertical distance from the TaperTipTM emitter to the sample surface, 2–3 mm; vertical distance between the bottom of the MS inlet and sample surface, 0–1 mm; and angle of the sprayer incidence, 55°.

### Data Acquisition, Processing and Analysis

Data acquisition, processing and reconstruction of imaging were performed using Masslynx 4.1 and High Definition Imagine (HDI) software (Waters, United States). Raw data were acquired within an *m/z* range of 100 to 1,200. The scan rate was 200 μm/s in both the horizontal direction (X) and vertical direction (Y) with one mass spectral scan per second in resolution mode. The pixel resolution was set by using a single value for the X and Y pixel size option of 200 μm. For 2D-MS imaging reconstruction, peak picking selected 1,000 of the most intensive peaks in the DESI experiment with an *m/z* range from 400 to 1,000 for PL profiling. The *m/z* window and resolution were the default settings of a 0.02 bin width and 20,000 resolution, respectively. To compare the imaging effectiveness acquired, the peak intensity was normalized by the internal standard LE (*m/z* 556.2720) at a concentration of 400 pg/μl and a flow rate of 5 μl/min continuously.

To obtain data containing *m/z* and peak intensity, region of interest (ROI) tool in HDI software was used to generated Waters .raw file. Then, Progenesis Bridge was utilized to transform Waters .raw file into direct sample data format. Subsequently, the direct sample data were imported into Progenesis QI version 2.3 (Nonlinear Dynamics, Waters, United Kingdom) for data preprocessing, including peak picking, and normalization as well as putative identification. The sensitivity of peak picking algorithm was set at automatic sensitivity method using noise estimation algorithm. Finally, normalized datasets containing sample description and peak intensity were imported SIMICA-P version 13.0 (Umetrics, Umea, Sweden) and Metaboanalyst^1^ to carry out statistical analyses, such as principal component analysis (PCA) and orthogonal partial least squares-discriminatory analysis (OPLS-DA) (Trygg et al., 2007). Putative identification from the PL database of detected features was performed on the basis of accurate mass (Fahy et al., 2005). Precursor tolerance was set at 5 ppm and fragment search method was selected with theoretical fragmentation at mass errors below 10 ppm. Moreover, the cleavage pattern of PLs from relevant published literatures (Hsu and Turk, 2009; Xia and Jemal, 2009; Pi et al., 2016) were used to corroborate the identification.

Univariate statistical analysis was carried out by Origin software (OriginLab, Northampton, MA, United States). Statistical significance was assessed by one-way analysis of variance (ANOVA) followed by Fisher’s least significant difference (LSD) test, and a *p*-value < 0.05 was considered significant.

## Results and Discussion

### Carrier Material

To assess the effects of carrier materials mentioned above, a number of PLs with relatively high signal-to-noise ratios were used. Further identification information is given in Supplementary Table S1. In this part of the experiment, a 10 μl volume of plasma was used for DPS preparation. ACN was used as the sprayer solvent with a flow rate of 10 μl/min. Plasma samples were analysed by scanning across each DPS with the aid of a 2D-moving stage.

**FIGURE 1:**
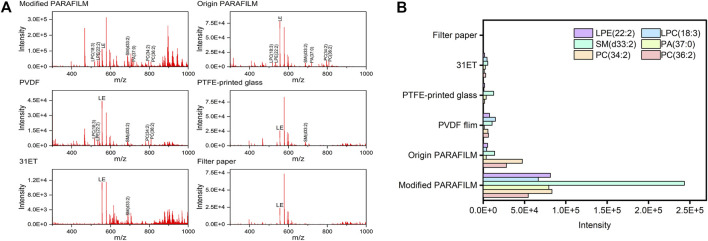
**(A)** Mass spectra obtained from DPSs on the six types of carrier materials **(B)** Total signal intensities of selected PLs on the six types of carrier materials. Abbreviations: LPE, lysophosphatidic ethanolamine; LPC, lysophosphatidylcholine; SM, sphingomyelin; PA, phosphatidic acid; PC, phosphatidylcholine.

As the mass spectra acquired from the strongest scan point on total ion chromatography of each material showed in Figure 1A, we observed that the whole signal intensities of compounds in non-cellulose-based carrier materials were commonly higher than those in cellulosed-based carrier materials. Thus, the accumulation of mass spectra of 10 scans were operated in order to detect the intensity easily (Figure 1B). However, once filter paper and DBS card were used as the carrier materials, the signal-to-noise of selected PLs still could not be detected effectively as well. This could be explained by the compounds being absorbed by the cellulose-based materials during the sampling procedure and obstructing their desorption. In addition, with the increase in pore size of the paper-based carrier, the diffusion rate increased, which in turn led to a decrease in the effective concentration per unit resolution area. In fact, when using non-cellulose-based carrier material, blood samples with a certain spatial volume were easily detached and sprayed away by nebulizing gas during DESI-MS operation; hence, it was difficult to acquire the complete analytes’ signal intensities from these DPSs. This “wash effect” (Pasilis et al., 2007) actually limited the application of non-cellulose-based materials in DESI-MS. To overcome this difficulty, we modified the shape of PARAFILM due to its extensibility; that is, we imprinted a certain number of circular grooves with 0.1 mm in depth and 4 mm in diameter in PARAFILM, on which DPSs could be fixed firmly in the course of DESI-MS analysis. As a result, the modified PARAFILM showed excellent signal enhancement compared with the other materials, which indicated that gas-phase analytes generated inside the grooves may have more adequate secondary extraction and ionization with highly charged microdroplets than those on the planar surface. Based on the above results, the modified PARAFILM was selected as the new carrier base for DPS sampling due to its weak sample−material interactions and optimal compound detective power.

### Sample Volume

**FIGURE 2:**
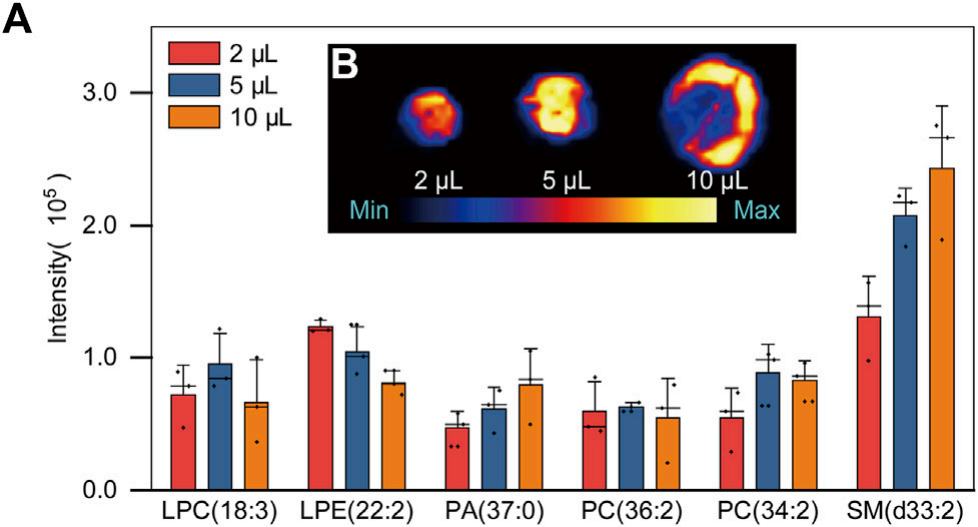
**(A)** Bar chart of PLs signal intensities in three different volumes of plasma samples: 2, 5 and 10 μl **(B)** Two-dimensional distribution diagram of SM (d33: 2) of DPSs in three different sample volumes.

In this section, the influence of the plasma droplet volume (2, 5 and 10 μl) on PARAFILM was studied. As shown in Figure 2A, there was no significant difference in signal among the sample volumes applied, which was consistent with previous reports (Fan et al., 2011; Richardson et al., 2018), and the reason was that the sample volumes were all relatively small (<20 μl). However, when the volume reached 10 μl, the distribution of plasma in the two-dimensional direction became obviously uneven, and SM(d33:2) was chosen to show the phenomenon of homogenization (Figure 2B). Normally, the greater the plasma volume is, the higher the concentration of compounds in the droplet. Therefore, the recommended sample volume used for further PL profiling was set to 5 μl in this work.

### Optimization of the Sprayer Solvent System

**FIGURE 3:**
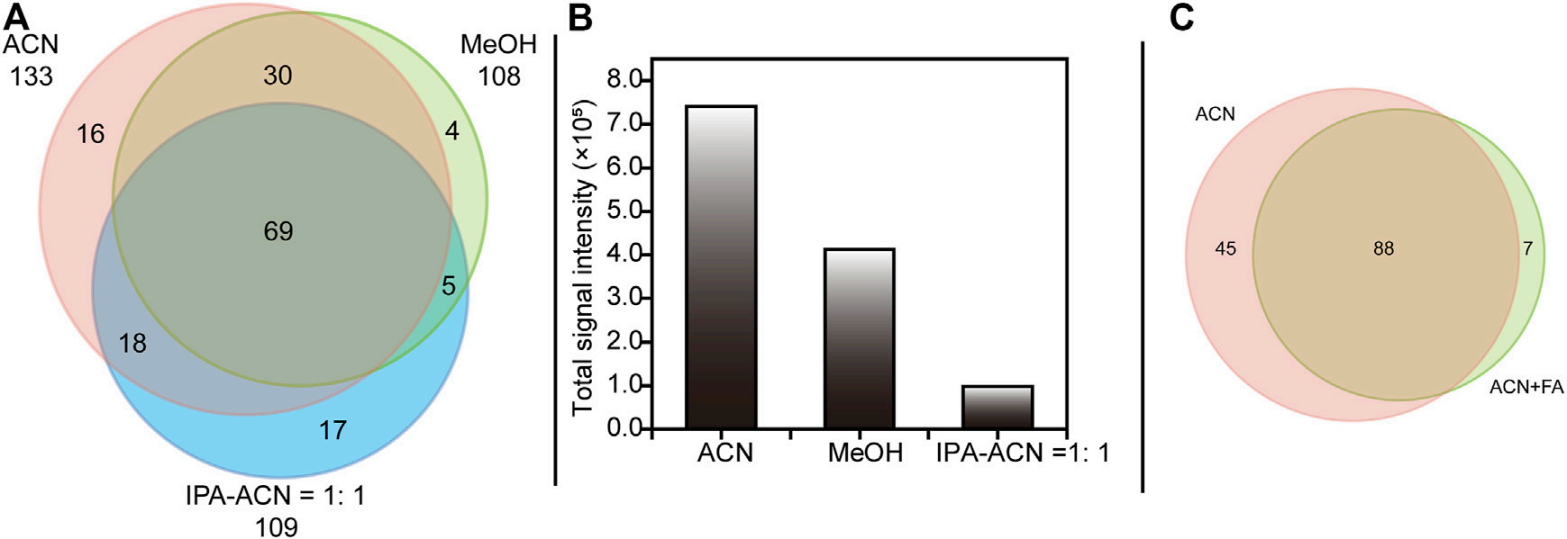
Venn diagram **(A)** shown the selectivity of PLs among ACN, MeOH and IPA-ACN = 1:1 and **(C)** shown the influence of addictive during DESI-MS analysis. Bar chart **(B)** showing the overall peak intensities of the putatively identified PLs. Abbreviations: MeOH, methanol; ACN, acetonitrile; IPA-ACN = 1:1, isopropanol-acetonitrile (1:1, V/V); FA, 0.1% formic acid.

Another critical factor for successful DESI-MS experiments in bioanalysis is the solvent system, including sprayer solvents and their additives. To date, various solvent systems (Manikandan et al., 2016) have been used for biological DESI-MS analysis, commonly containing various ratios of MeOH, ACN and water. For PL profiling, three different solvent systems, MeOH, ACN, and IPA-ACN (1:1, V/V), were tailored in our experiment based on the properties of PLs. The Venn diagram in Figure 3A summarizes the coverage of PLs in each sprayer solvent system and shows that ACN exhibited maximum PL coverage. Furthermore, as demonstrated in Figure 3B, a remarkable increase in total signal intensity was observed when using ACN instead of IPA-ACN (1:1, V/V) in this experiment. These results might be due to the high viscosity of IPA (0.310 mPa·s), and the “pickup” of PLs in those groups containing IPA was less than that in groups containing only ACN (-0.467 mPa·s) (Kauffman and Jurs, 2001; Venter et al., 2006). In addition, the PLs selectivity was decreased when employing ACN with 0.1% FA as the sprayer solvent, indicating that the presence of FA may inhibit the ionization of some PLs (Figure 3C). According to the acquired results, we selected ACN as the sprayer solvent in DESI-MS since it provided the best coverage and overall signal intensity for PLs in this study.

Thus far, a PL profiling method with DPS sampling and a DESI-MS instrument has been established based on the results obtained above. Namely, a 5-μl volume of plasma was deposited on a modified PARAFILM substrate and then desorbed by using ACN as the sprayer solvent for DESI-MS analysis.

### Stability and Repeatability

To achieve accurate phospholipidomics analysis, it is important to ensure that the DESI-MS system is robust against variation in certain parameters such as signal intensity during operation. However, systematic deviation of ion intensity will occur in the course of acquisition due to slight changes in the spray conditions. Hence, LE was utilized as an internal standard to correct this variation. DESI-MS analysis requires samples to be stable at room temperature and atmospheric pressure (Ganz et al., 2012). Therefore, the stability of PLs in the DPSs on modified PARAFILM was assessed in the following experiment. We selected several time points (6, 24, 48, 72 and 168 h) after dried spots formed at room temperature in a desiccator for 24 h to perform the stability tests. All samples were kept in a desiccator during the storage period at room temperature.

**FIGURE 4:**
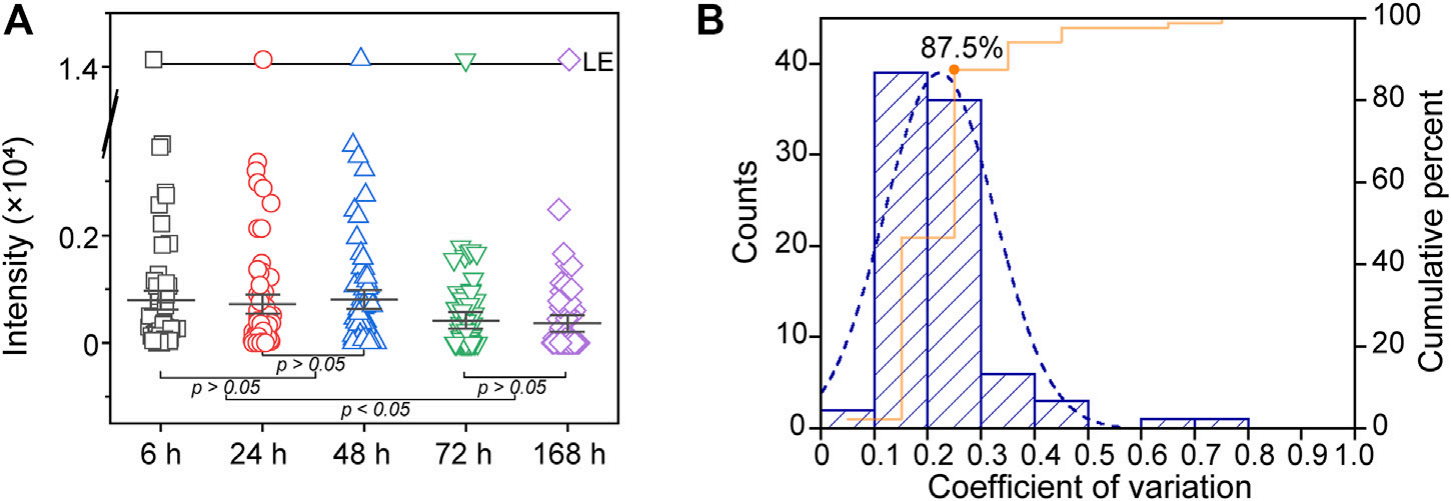
**(A)** Several time points (6, 24, 48, 72 and 168 h) were used to evaluate the stability of PLs in DPSs on modified PARAFILM **(B)** Histogram of the coefficient of variation (CV) demonstrating the repeatability of the established method.

The differences in signal among groups were obvious at the 0.05 level (one-way ANOVA), indicating that the total signal of PLs dynamically changed during the specified time period (from 6 to 168 h). The overall mean PL was significantly lower at 72 and 168 h than at 6, 24 and 48 h (*p* < 0.05). Additionally, there was no significant difference between the 6, 24 and 48 h groups (*p* > 0.05, Figure 4A). Therefore, we suggest the operating time of DESI-MS analysis should less than 48 h to ensure the reliability of the data.

Furthermore, six repeated dried spots (each spot containing 5 μL of plasma) were pipetted onto modified PARAFILM and allowed to dry for 24 h in the desiccator at room temperature. The coefficient of variation (CV) of PLs calculated from the above DPS samples was used to evaluate the repeatability of the method (Figure 4B). The CV distribution of PLs in this study showed that the DESI-MS system and DPS samples were consistent over the analysis duration. Indeed, we observed that the percentage of PLs was 87.5% (CV% < 30), proving that applying modified PARAFILM as a DPS sampling carrier for phospholipidomics was feasible (Gika et al., 2007).

### Method Application

The above fit-for-purpose, optimized experiments demonstrated the promising combination of PARAFILM-based DPS sampling and DESI-MS analysis. Therefore, an *S. japonicum* infection model was established and employed to evaluate the classification potential of DPS sampling DESI-MS with optimal conditions in a phospholipidomics study. The procedural blank control sample containing system noise and impurity data from the carrier and instrument, but without a plasma sample, was also acquired to eliminate system deviation.

**FIGURE 5:**
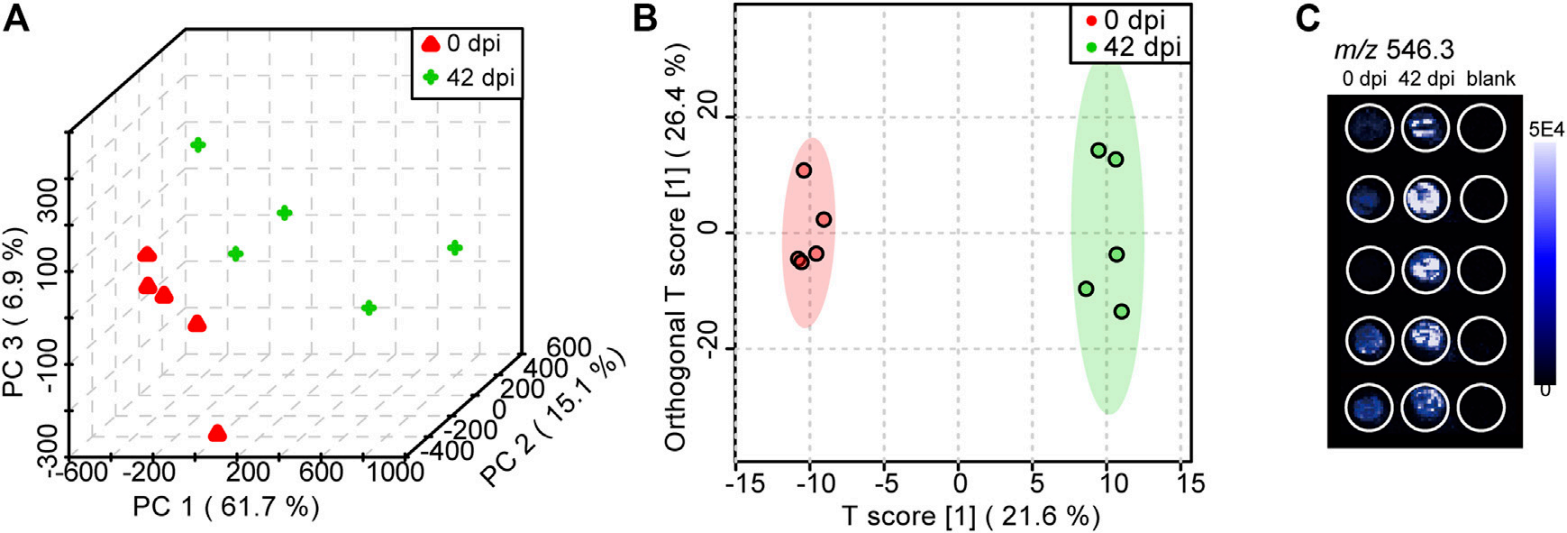
PCA **(A)** and OPLS-DA **(B)** score plots obtained from the *Schistosoma japonicum* (*S. japonicum*)-infected group (42 dpi) and control group (0 dpi) using DPS sampling with DESI-MS analysis **(C)** A DESI-MS image of the concentration status of LysoPC (22:3) (*m/z* 546.3) in different groups.

The PCA score plot (Figure 5A) and OPLS-DA score plot (Figure 5B) showed well-distinguished clusters between the control group and infected group, indicating that *S. japonicum* infection led to PL alterations in the plasma samples of mice. PCA is an unsupervised multivariate pattern recognition analysis method focusing on the overall clustering patterns and trends in a data set, while OPLS-DA is a supervised model for biomarker finding (Trygg et al., 2007). For supervised methods, it is very important to avoid overfitting of the data, so we applied the goodness of fit (R^2^Y) and the predictive ability (Q^2^Y) of the model to evaluate its quality. Normally, Q^2^Y values >0.5 are considered good for biological models (Broadhurst and Kell, 2006). In our OPLS-DA model, the values of R^2^Y and Q^2^Y were 0.994 and 0.693, respectively, demonstrating excellent fitness and predictability. Moreover, a permutation test (n = 1,000) was performed, and the *p*-value of Q^2^Y was less than 0.05. Above all, both the R^2^Y and Q^2^Y values and the permutation test illustrated the high reliability of this OPLS-DA model. Furthermore, LysoPC (22:3) (*m/z* 546.3) was selected as a discriminated metabolite between healthy mice and *S. japonicum*-infected mice, based on the variable importance in projection (VIP) score that taken from comparison in OPLS-DA model >2 and *p*-value from student’s *t*-test < 0.05. The mass spectral image, depending on DESI-MS imagine technology, reconstructed by HDI software showed that the concentration of LysoPC (22:3) (VIP = 5.6, *p* = 0.009) in the plasma of mice increased significantly at 42 dpi compared to 0 dpi (Figure 5C). LysoPC plays an important role in regulating the immune response, and a high level of LysoPC in the plasma is regarded as a marker for cell membrane injury (Hu et al., 2020). These results indicated that PARAFILM-based DPS sampling with DESI-MS technology is promising for rapid untargeted lipidomics screening. More meaningfully, as discussed in Kauppila et al. (2007), selectivity in the DESI-MS platform for different kinds of analytes depended on the spray solvent composition. Therefore, based on the property of polar metabolites, a combination of water with MeOH would be the better solvent system, which indicated that this study also lay the foundation for rapid metabolomics screening research.

## Conclusion

To date, using DESI-MS to analyse DPS directly is relatively rare because there is no proper carrier substrate for blood samples. In this study, to overcome the obstacle of combining DPS with DESI-MS, six types of materials, including non-cellulose-based (modified PARAFILM and original PARAFILM, PVDF, and PTFE-printed glass slides) and cellulose-based (Whatman 31 ET and filter paper) materials were evaluated. Due to the strong absorption of 31 ET and filter paper, the PLs were not sufficiently detected. Furthermore, we overcame the challenge of the “wash effect” using a modified PARAFILM, which exhibited excellent signal enhancement. Based on this result, the modified PARAFILM was selected as a new carrier base for DPS sampling. Then, a rapid phospholipidomics approach was successfully established by evaluating the sampling volume and sprayer solvent system. Therefore, 5 μl of plasma samples were deposited on the PARAFILM carriers with ACN as the sprayer solvent in DESI-MS analysis. This method performed quite well in stability and repeatability tests and could be employed in the untargeted metabolomics study of schistosomiasis.

The PARAFILM-based DPS sampling and DESI-MS analytical method we developed and validated not only exhibited sufficiently high signal intensities of compounds but also had great advantages in terms of simplicity, cost, and acquisition. The described method presented a high-throughput analytical means for PL profiling of blood samples compared to conventional liquid chromatography coupled with mass spectrometry. In summary, the promising results of this study lay the foundation for rapid metabolomics screening research and are amenable to clinical studies. Nevertheless, further investigations on the benefit of PL profiling with the PARAFILM-based DPS sampling and DESI-MS method should be initiated to determine whether this method could achieve the required precision of medicinal and population health studies.

## Data Availability

The raw data supporting the conclusions of this article will be made available by the authors, without undue reservation.

## Footnotes

1
https://www.metaboanalyst.ca/
