# Supplementary material for: The Imprinted PARAFILM as a New Carrier Material for Dried Plasma Spots (DPSs) Utilizing Desorption Electrospray Ionization Mass Spectrometry (DESI-MS) in Phospholipidomics
Source: Front Chem. 2021 Dec 10;9:801043. doi: 10.3389/fchem.2021.801043 (PMC8702624; doi:10.3389/fchem.2021.801043)
Supplement: Supplementary file 1 [file Table1.DOCX]

Supplementary Table 1 Identification information of selected PLs

| *m/z* | MS/MS | Identifier |
| --- | --- | --- |
| 518.3223 | 518.3, 459.2, 184.0 | LPC (18:3) |
| 534.2960 | 534.2960^*^ | LPE (22:2) |
| 687.5477 | 687.5, 184.0 | SM (d33:2) |
| 719.5522 | 719.5, 619.4, 441.3 | PA (37:0) |
| 796.5245 | 796.5, 737.4, 613.4, 534.3, 184.0 | PC (34:2) |
| 808.5802 | 808.5, 749.4, 625.4, 546.3, 184.0 | PC (36:2) |

^*^Presents only accurate mass spectral data. Abbreviations: LPC, lysophosphatidylcholine; LPE, lysophosphatidyl ethanolamine; SM, sphingomyeline; PA, phosphatidic acid; PC, phosphatidylcholine.
