# Supplementary figures and images for: The Imprinted PARAFILM as a New Carrier Material for Dried Plasma Spots (DPSs) Utilizing Desorption Electrospray Ionization Mass Spectrometry (DESI-MS) in Phospholipidomics
Source: Front Chem. 2021 Dec 10;9:801043. doi: 10.3389/fchem.2021.801043 (PMC8702624; doi:10.3389/fchem.2021.801043)

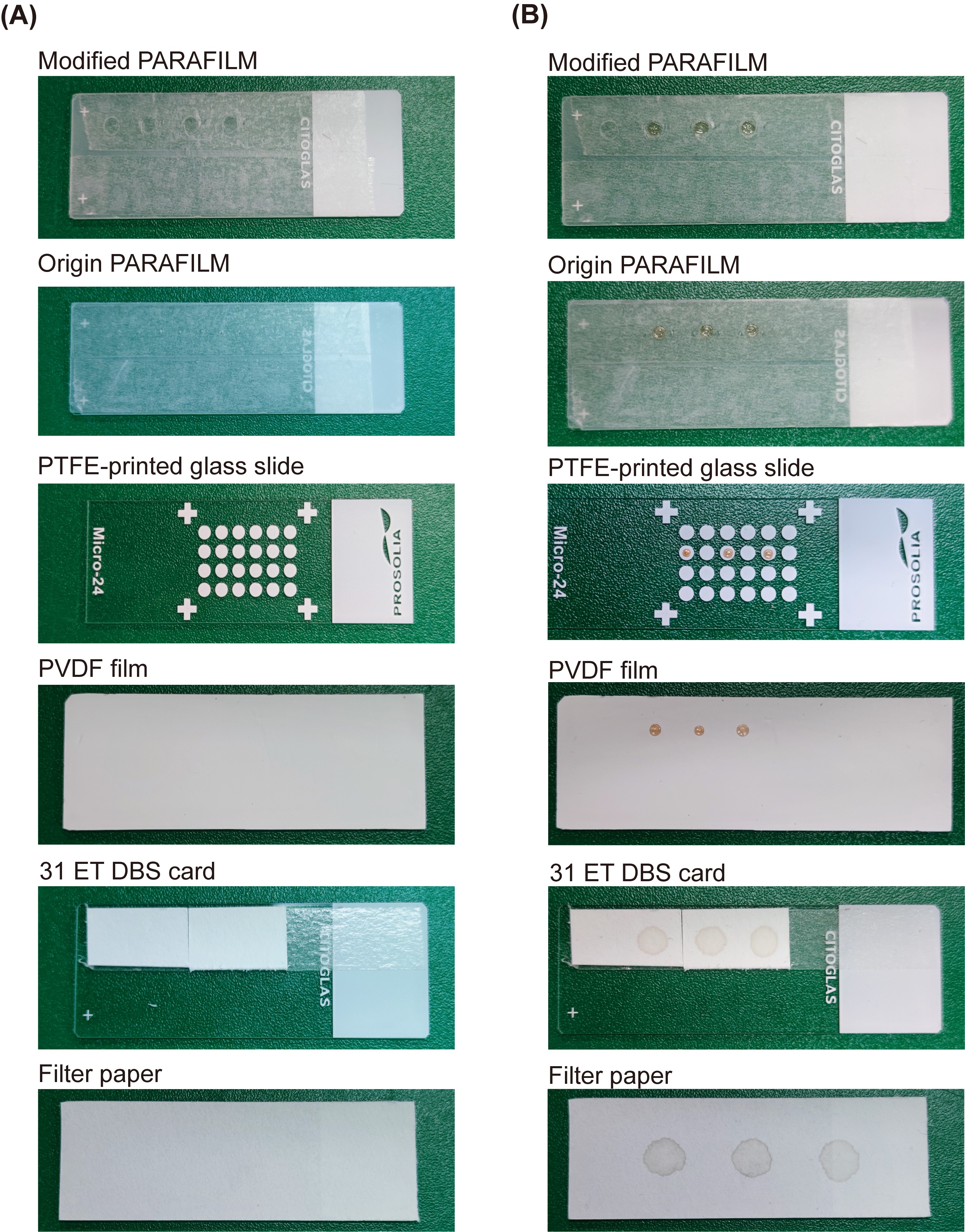

Supplement: Supplementary file 2 [file Image1.JPEG]

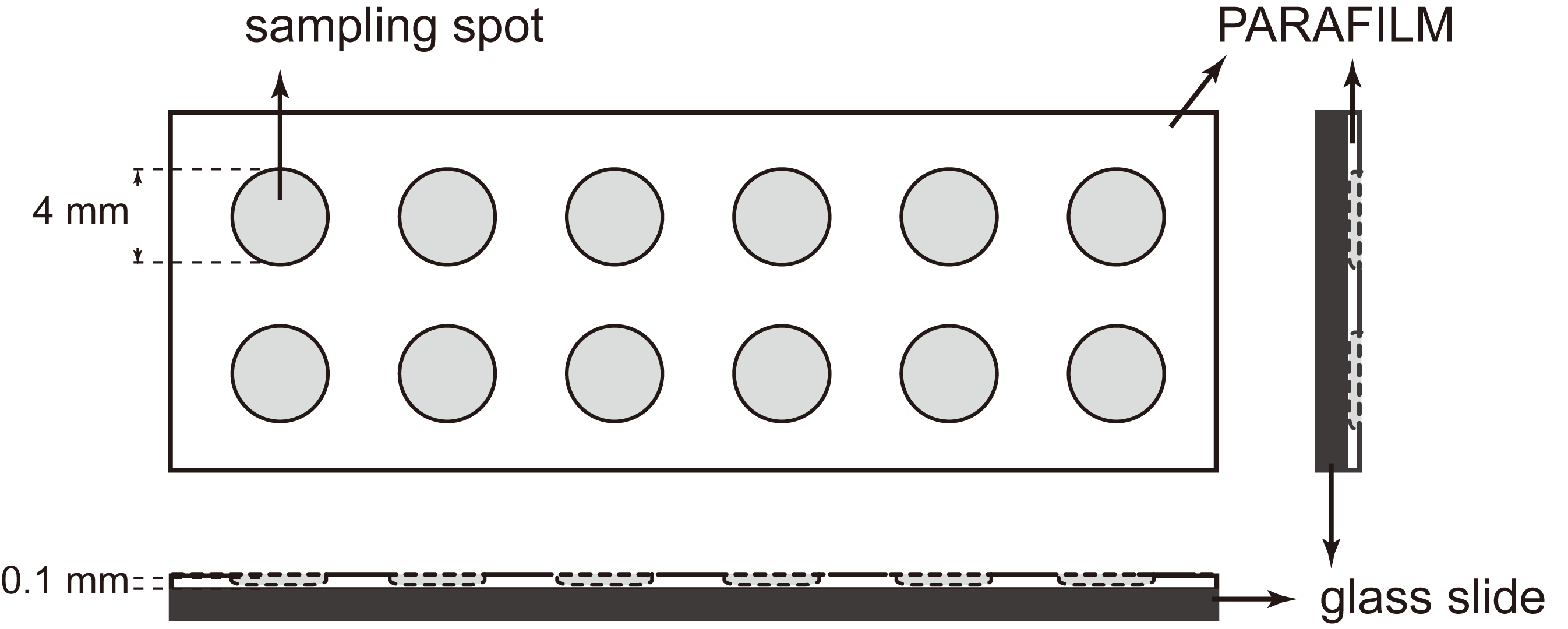

Supplement: Supplementary file 3 [file Image2.TIF]
